# Supplementary material for: Polyketide synthase-derived sphingolipids mediate microbiota protection against a bacterial pathogen in C. elegans
Source: Nat Commun. 2025 Jun 3;16:5151. doi: 10.1038/s41467-025-60234-1 (PMC12134224; doi:10.1038/s41467-025-60234-1)
Supplement: Supplementary file 1 — Supplementary Information [file 41467_2025_60234_MOESM1_ESM.pdf]

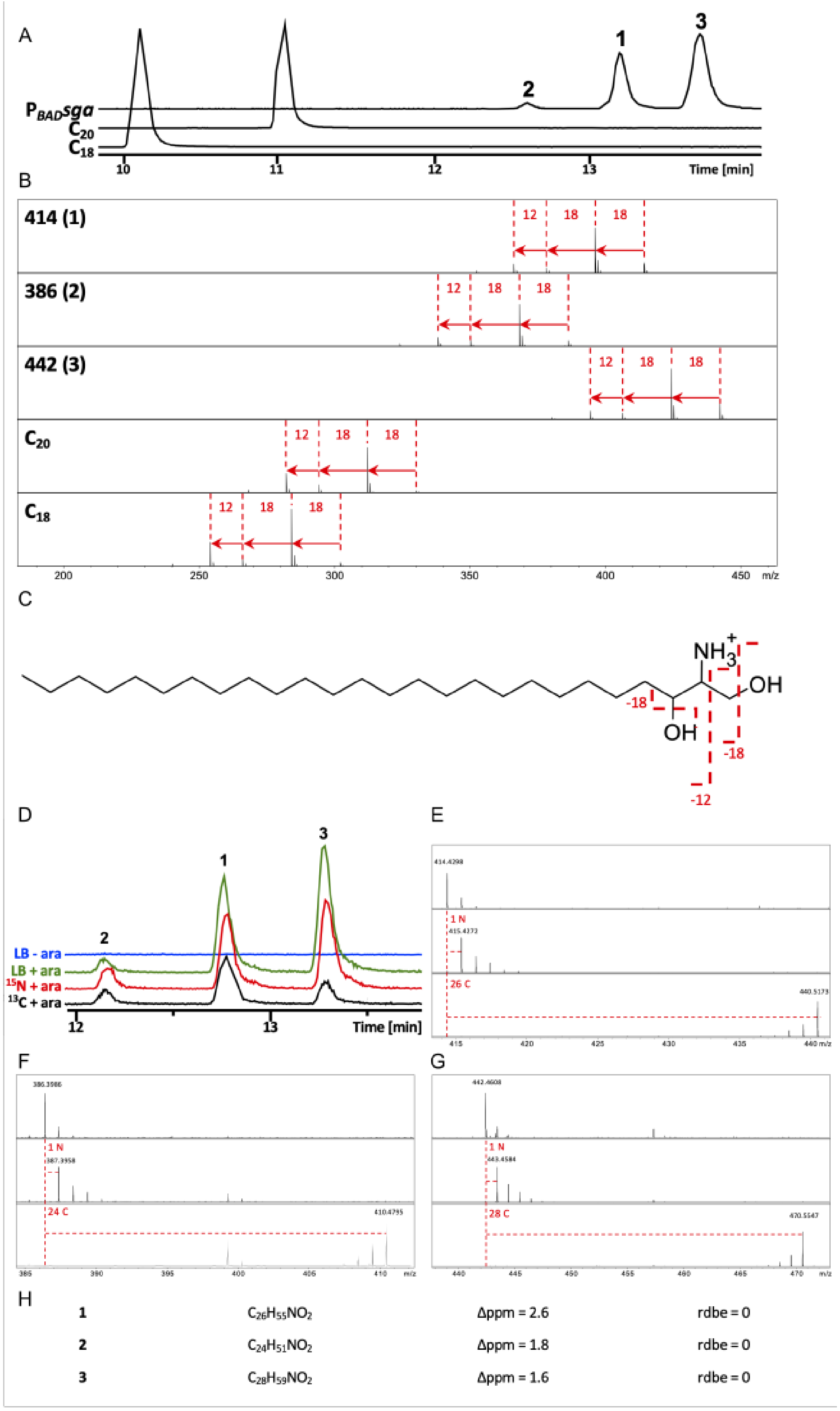

Figure S1: MYb115 PKS SgaAB-derived compounds 1-3 are very long chain sphinganines. (A) Extracted ion chromatograms of compounds **1**, **2** and **3**, as well as sphinganine (d18:0) and sphinganine (d20:0). **(B)** Fragmentation patterns of **1**, **2** and **3**, as well as sphinganine (d18:0) and sphinganine (d20:0). **(C)** Fragmentation of sphinganines, exemplary shown for the structure of compound **1**. **(D-H)** Sum formula determination of compounds **1**, **2** and **3** using isotopic labelling and LC-MS. **(D)**: Extracted ion chromatogram (EICs) of compounds **1**, **2** and **3** in unlabelled samples with (green) and without arabinose (blue), as well as ^{15}N- (red) and ^{13}C-labelled (black) samples. **(E-G)**: Mass shifts compared to LB cultivation (dashed red lines) represent the number of carbon and nitrogen atoms incorporated. **(H)** Sum formula and structural data of compounds **1-3**.

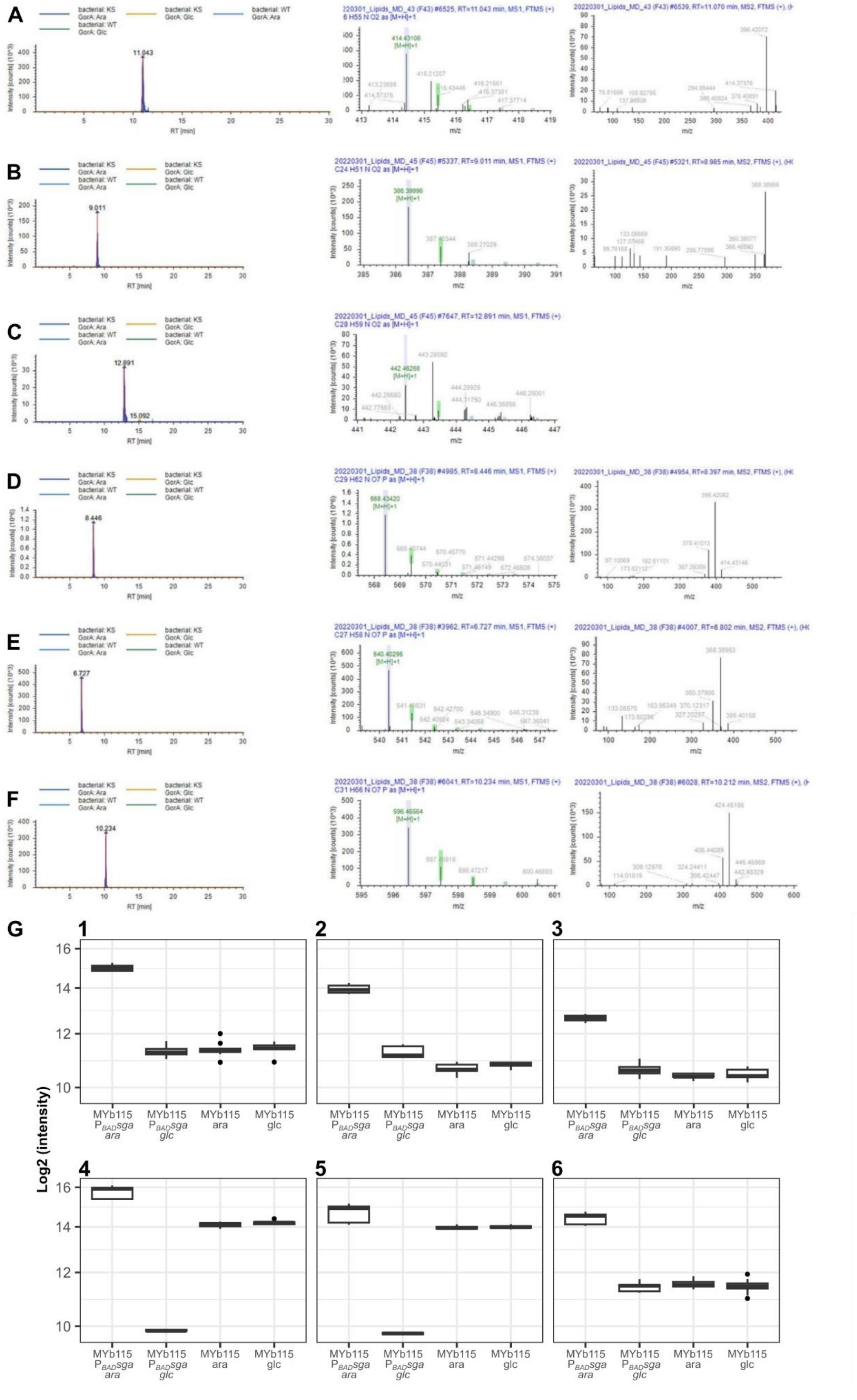

Figure S2: *P. fluorescens* MYb115 PKS produces long chain sphinganines and phosphoglycerol sphingolipids

Original LC-MS data of compounds **1** (**A**), **2** (**B**), **3** (**C**), **4** (**D**), **5** (**E**) and **6** (**F**) stemming from the lipidomics experiments. Left: LC chromatogram; Middle: MS spectra; Right: MS^2 spectra. Data was extracted using Compound Discoverer 3.3. **(G)** Relative abundance of the sphinganine compounds **1**, **2** and **3** and the PG- sphingolipids **4**, **5** and **6** in MYb115 wt and MYb115 P_{BAD}*sga* in the presence of arabinose (ara) for activation or in the presence of glucose (glc) for repression of transcription of P_{BAD}*sga.*

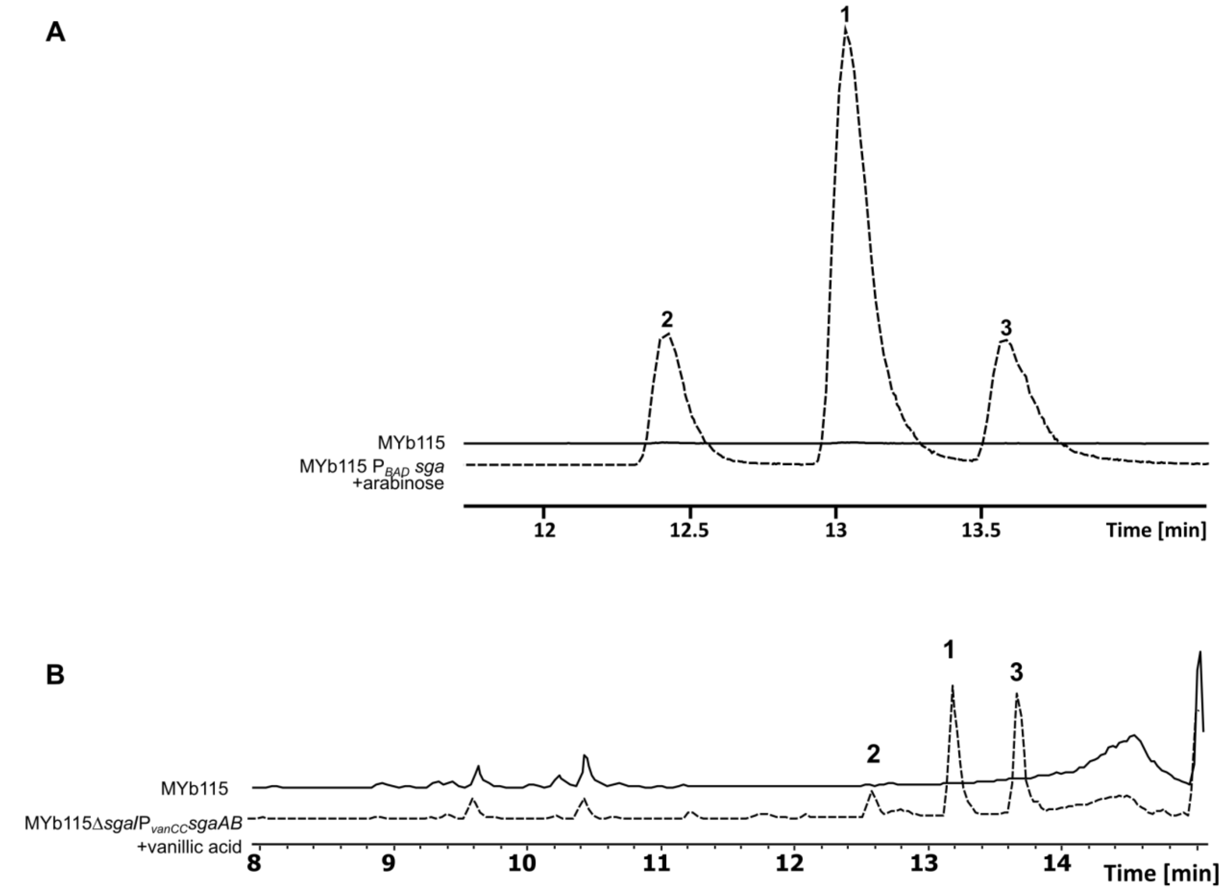

Figure S3: Induction of MYb115 P$_{BAD}$*sga* and MYb115 Δ*sgaA*/P$_{vanCC}$*sgaAB* leads to increased SL production. (A) LC-MS chromatogram comparing SL profiles of MYb115 wt (solid line) and MYb115 P$_{BAD}$*sga* induced with arabinose (dashed line). **(B)** LC-MS chromatogram comparing SL profiles of MYb115 wt (solid line) and MYb115 Δ*sgaA*/P$_{vanCC}$*sgaAB* induced with vanillic acid (dashed line). Upon induction with arabinose or vanillic acid, three distinct compounds **(1–3)** accumulate at much higher levels compared to the wild type.

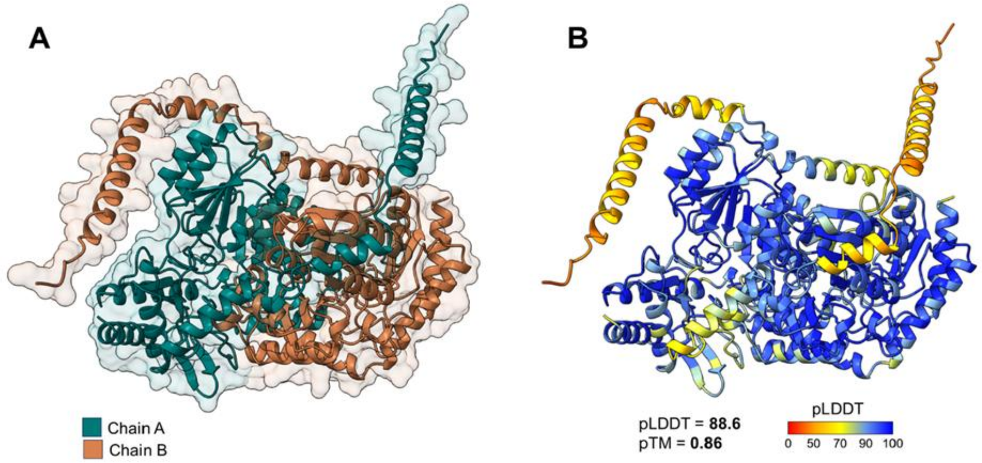

Figure S4: Alpha Fold3 model of *Pf*SgaB coloured by chain (A) and positional Predicted Local Distance Difference Test (pLDDT) scores, from 0-100 (B).

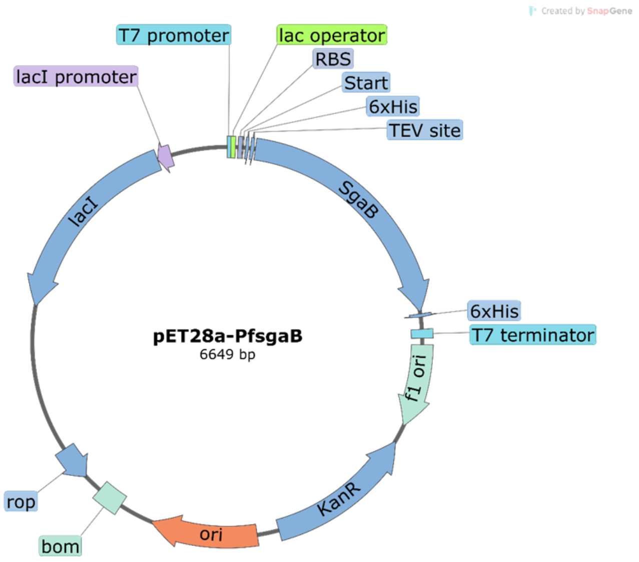

Figure S5: Annotated plasmid map of pET28a-*PfsgaB*

MGSSHHHHHHSSGENLYFQGHRTVKTPTGLSVGIKQRLIQQALERRRSEVGDAAGGATLEQAQQAALKVPEAFYRFDLHPGYQQLRVMQQGAARLG
LSSPFFRLHEGLAGAETRIGAQQYVNFASYNYLGYSGHPDVAEAAKAAIDRYGTSVSASRLVSGDRPLHRELERELAKLYEVDDAIVFVSGHATNVTTIG
HLFGPRDLVLHDELIHNSVLQGIQLSGARRLSFAHNDWQALDRILGEQRQHFERVLVVVEGIYSMDGDYPDLPRFVDIKRKHKTFLMVDEAHSLGVMGA
TGKGIREHFSLAGDDVDIWMGTLSKTLASCGGYIAGNTALVEHLKFLAPGFLYSVGMPPSTTASALAALRCLANDRERVQTVQARGELFLRLAKAAGLD
TATSTGLAVIPVITGSSFKAGRLSSALFERGINAQPILYPAVPEHVARVRFFVSCEHTEEQIRQTVAIVAEELAKIG*

Figure S6: Amino acid sequence of recombinant *Pf*SgaB. The N-terminal pre-sequence, encoding the N-terminal polyhiststidine tag and TEV cleavage site (underlined), is shown in **bold**.(*) indicates the presence of a stop codon (TAA).

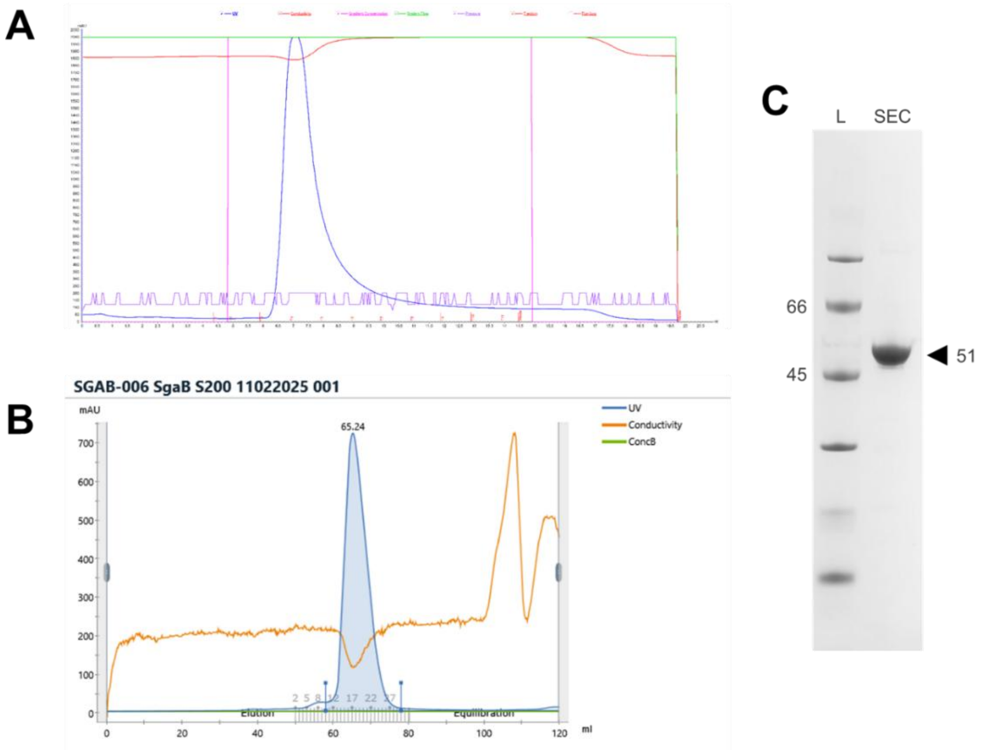

Figure S7: (A) *Pf*SgaB IMAC purification chromatogram using a HiTrap TALON Crude (1 mL). **(B)** *Pf*SgaB SEC purification chromatogram using a HiLoad 16/600 Superdex 200 pg (120 mL) column. (**C**) SDS-PAGE analysis of SEC-purified *Pf*SgaB. An Amersham Low Molecular Weight Calibration Kit was used as the protein ladder (lane L).

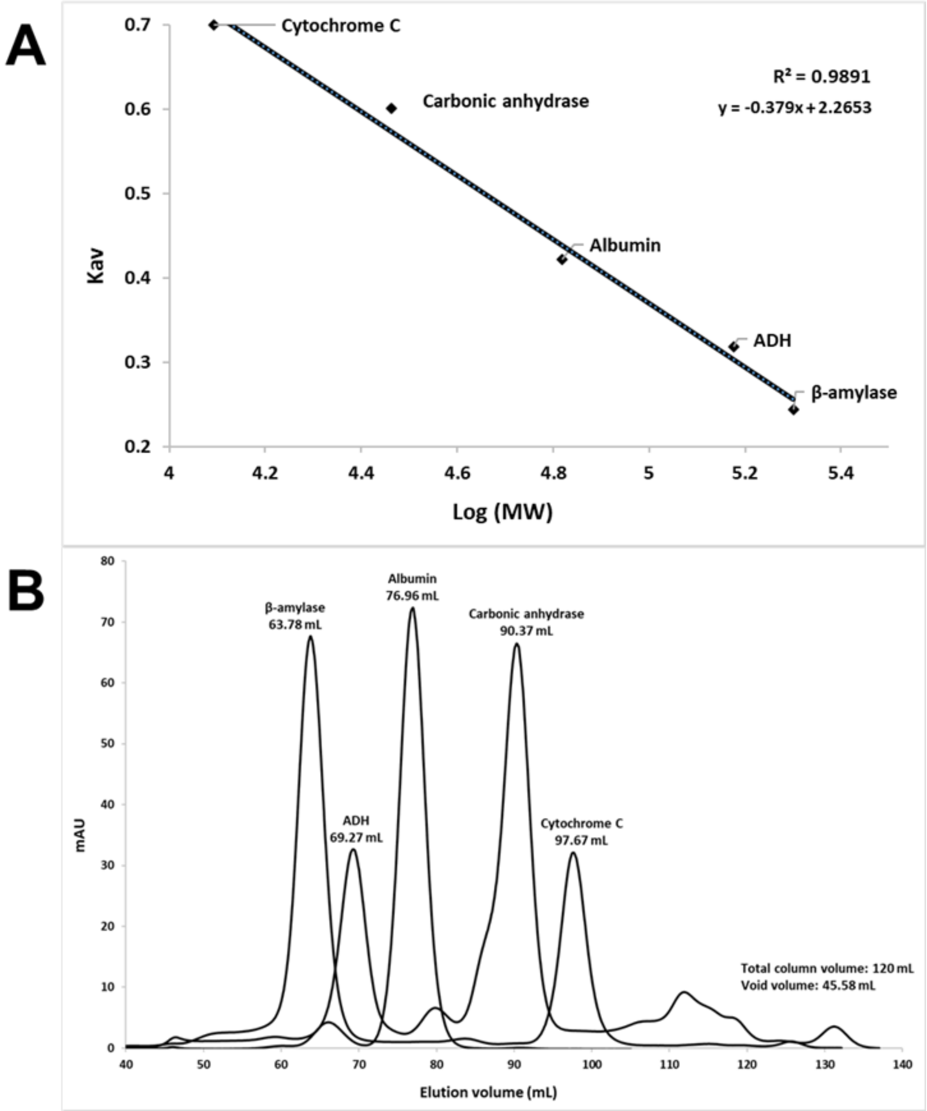

Figure S8: (A) HiLoad 16/600 Superdex 200 pg SEC calibration curve using commercial protein calibration standards. The *Pf*SgaB partition coefficient (K_{av}) was calculated using the following formula:

$$K_{av} = \frac{(V_e + V_i) - V_o}{V_t - V_o}$$

Where V_e = elution volume (65.24 mL); V_i = sample injection volume (8 mL); V_o = void volume (45.58 mL); and V_t = total column volume (120 mL). Using the trendline equation, the inverse log of the MW was calculated to be ~107.6 kDa, or 2.09X the monomeric SgaB MW, indicating protein dimerisation. **(B)** Overlaid SEC chromatograms of the protein calibration standards used in this study.

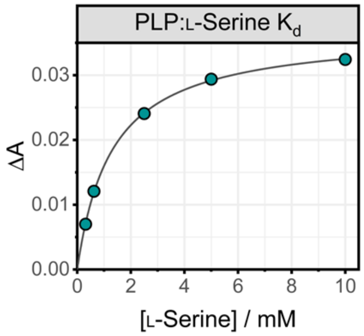

Figure S9: Fitted PLP:L-Ser binding curve (K_d).

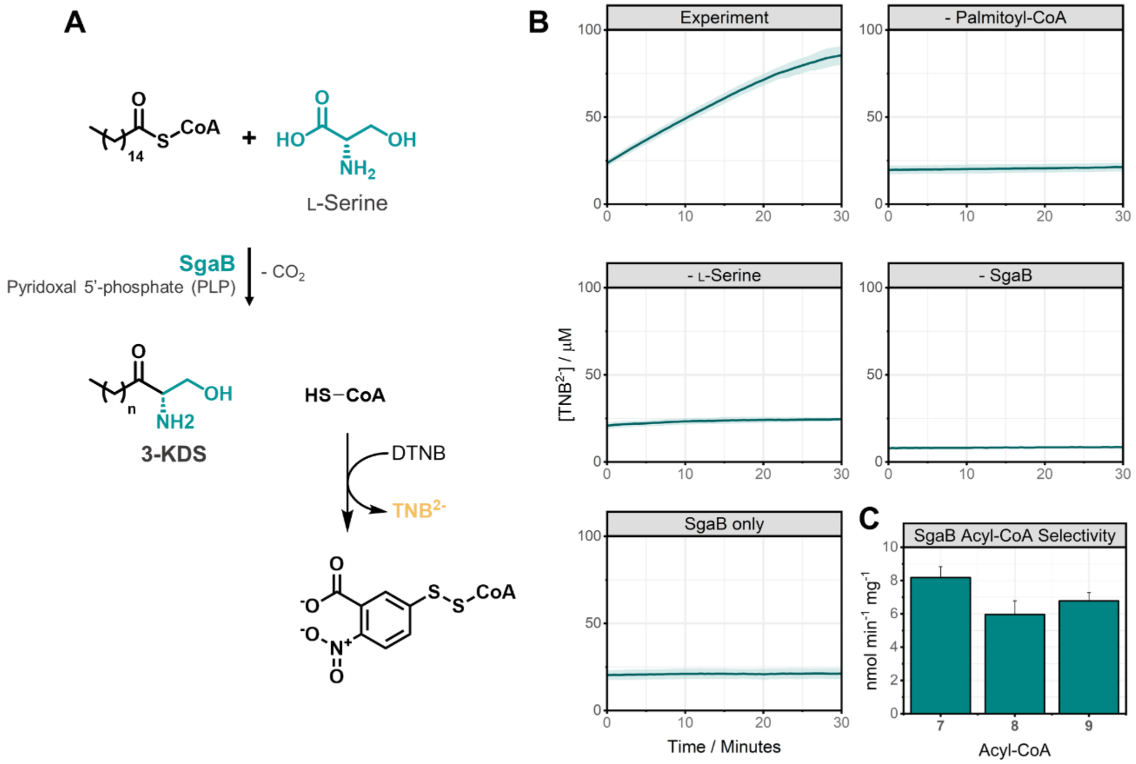

Figure S10: (**A**) DTNB assay scheme. The *Pf*SgaB-catalysed condensation of palmitoyl-CoA and L-serine releases free CoA (CoASH), which can react with DTNB to give a mixed disulfide. The released TNB^{2-} anion gives a characteristic yellow colour (λ_{max} = 412 nm). (**B**) *Pf*SgaB activity detected using the DTNB assay, monitored over 30 minutes. Shaded error bands represent the standard deviation of three technical replicates. (C) Measured *Pf*SgaB specific activity (t = 20 minutes) using L-serine and acyl-CoAs **7-9** as surrogate substrates, determined using the DTNB assay (412 nm). Error bars represent the standard deviation of three technical replicates. All measurements were corrected for non-specific background absorbance.

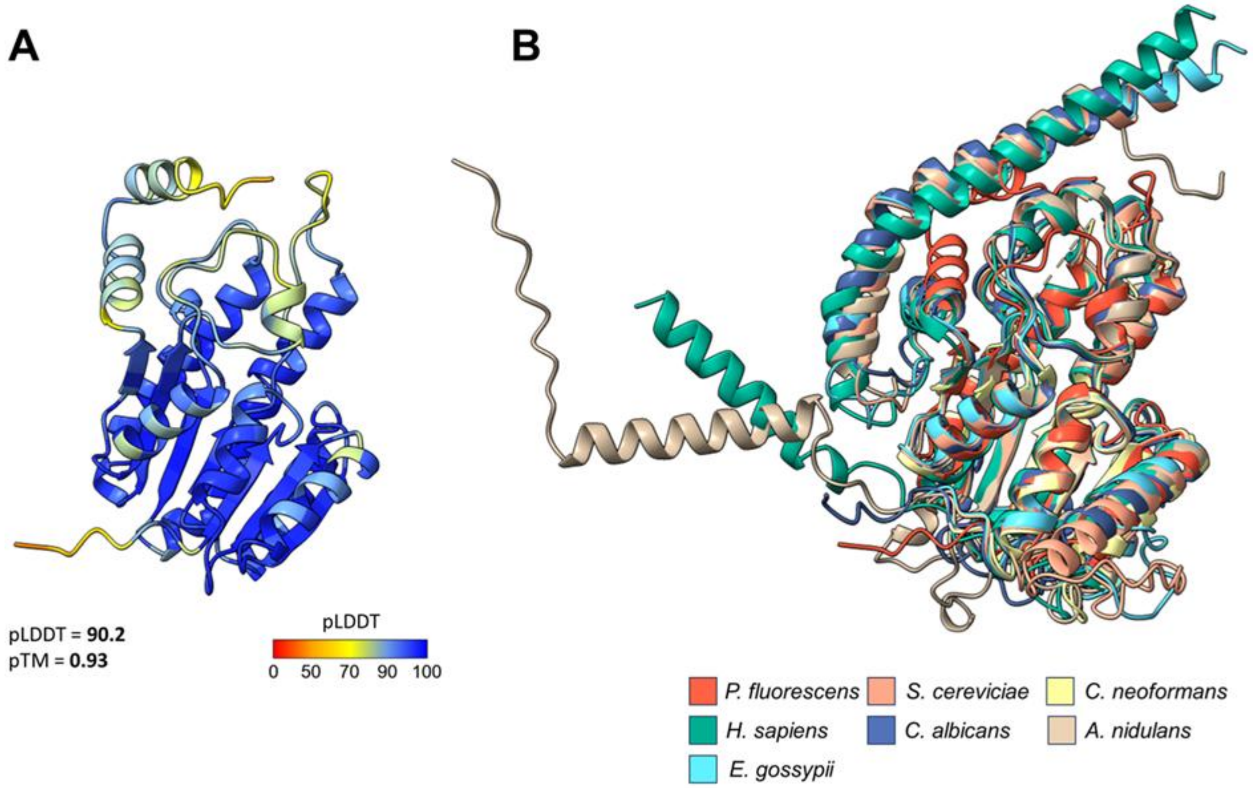

Figure S11: (A) AlphaFold3 prediction of *Pf*SgaC, the identified KDSR homologue in *P. fluorescens* (locus ID: KW062_RS19775), coloured by pLDDT. **(B)** Structural alignment of *P. fluorescens* putative KDSR with eukaryotic KDSR homologues (Uniprot: P38342, Q59RQ2, Q5BE65, Q758B6, Q06136, RMSD = 1.04-1.08 Å). All structures except *C. neoformans* KDSR (PDB: 8JAT) were retrieved from AlphaFold database.

```
Pseudomonas_fluorescens      1  .........................................MTQHSSPRCILITGATGGI
Aspergillus_nidulans         1  MHPSLPSIIYDASPTALGISAVFGALFFYTLVKMFGFLARENQFVVEGRTVVITGGSEGM
Candida_albicans             1  ....................................MWFSKTNFPVEGKTALIVGASQGI
Saccharomyces_cerevisiae     1  .........................................MKFTLEDQVVLITGGSQGL
Eremothecium_gossypii        1  .........................................MKYELNGQVVLISGGSQGL
Homo_sapiens                 1  ...............MLLLAAAFL.VAFVLLLYMVSPLISPKPLALPGAHVVVTGGSSGI

Pseudomonas_fluorescens     20  GGALAPAYAAPGVTL..ILQGRRLDRLEEMASECRAL.......................
Aspergillus_nidulans        61  GKAVACQLAQKGANI..VIVARTLQKLEEAIEAIKGSA..................ANVN
Candida_albicans            25  GVNLAERLYEKNCSTI..LVARTESKLQHQIQNIKEK...................YPES
Saccharomyces_cerevisiae    20  GKEFAKKYYNEAENTKIIIVSRSEARLLDTCNEIRIEAHLRRETTDEGQVQHKLAAPLDL
Eremothecium_gossypii       20  GRAFAQKYIEESDSTV.VIVSRSEEKLTRAGEAICGG...............ARRLGAGG
Homo_sapiens                45  GKCIAIECYKQGAFI..TLVARNEDKLLQAKKEIEMHS...................IND

Pseudomonas_fluorescens     55  GARVLLEALDVR.DLDALRAMVRRVS...EAEQPDLVLVGAGLNTAVGSNGEAEDWDASC
Aspergillus_nidulans       101  KQRFHYISADLT.KPEECERIMTEVTEWNDGMPPDIVWCCAGYCTPGYFVET..SVQTLK
Candida_albicans            64  SAKISYAVADVS.NYDECTRLWRTID..PA..DPDILFCCAGSSIPKLFQDL..TKVDIE
Saccharomyces_cerevisiae    80  EQRLFYYPCDLS.CYESVECLFNALR..DLDLLPTQTLCCAGGAVPKLFRGL..SGHELN
Eremothecium_gossypii       64  AGRLLYYACNLG.DAAAVGGLFATLA..DAGLQVTQVLFCAGGAVPGLFAEL..SSAQLA
Homo_sapiens                84  KQVVLCISVDVSQDYNQVENVIKQAQ..EKLGPVDMLVNCAGMAVSGKFEDL..EVSTFE

Pseudomonas_fluorescens    111  ALLEVNVMAALATVDAALP..........VMRARGHGQIALFSSLAGWRGLPVTPTYSAS
Aspergillus_nidulans       158  DQMDTVYWTAANTAHAILRKWLVPIN.PSHQRPLPRRHLIFTCSTLAFVPIAGYAPYSPA
Candida_albicans           117  SGIDINYKTVINVVHTGFKHALSNNTDNLEPHNFKKRSVVLFSSVVSFFPFIGYSQYAPM
Saccharomyces_cerevisiae   135  LGMDINYKTTLNVAHQIAL...........AEQTKEHHLIIFSSATALYPFVGYSQYAPA
Eremothecium_gossypii      119  AGVEMNYGTALHLAHGAV..............RHGARHLVFFSSAAAVYPFIGYSQYAPL
Homo_sapiens               140  RLMSINYLGSVYPSRAVITT..........MKERRVGRIVFVSSQAGQLGLFGFTAYSAS

Pseudomonas_fluorescens    161  KAAIRVYGEAIRDWLAPE...............GVKINVILPGYVESKMCFEMPGPKPFL
Aspergillus_nidulans       217  KAAMRALSDTLCQEIEVYNGSRASKERARATPADVKIHTVFPMGILSPGFDNEQQIKPAL
Candida_albicans           177  KSAIESLSIILRQELSPY...............NYRVTCVFPGNFQSEGFEEEQKTKPDI
Saccharomyces_cerevisiae   184  KAAIKSLVAILRQELT.................NFRISCVYPGNFESEGFTVEQLTKPEI
Eremothecium_gossypii      165  KAALRALVAVLRQECD.................GVRVSCVYPGNFASEGYAEENRTKPAI
Homo_sapiens               190  KFAIRGLAEALQMEVKPY...............NVYITVAYPPDTDTPGFAEENRTKPLE

Pseudomonas_fluorescens    206  WT..........AEKAARRIKRGLAANQARISFPFP...................LNLGT
Aspergillus_nidulans       277  TKQLESADKPQTPKEVARIAIEAIERGEYLITTMFVGDVMKGAALGPSPRNSWFRDTCTG
Candida_albicans           222  TKKIEGPSNPIPGDECARLIIDQLDKGYDSITTDFIGWFLGCSVLGISS........PRQ
Saccharomyces_cerevisiae   227  TKLIEGPSDAIPCKQACDIIAKSLARGDDDVFTDFVGWMIMGMDLGLTAKKS..RFVPLQ
Eremothecium_gossypii      208  TAAIEGSSEAISCAACCDKIVRGLRSGYDDVTTDFVGWLLLACNMGFNYHSTTYFLWPLG
Homo_sapiens               235  TRLISETTSVCKPEQVAKQIVKDAIQGNFNSSLGSDGYMLSALTCGMAPVTSITEGLQQV

Pseudomonas_fluorescens    237  WLLGVIPQRLS.SFILRGLNYSE...................
Aspergillus_nidulans       337  WLSNLLFLGVVPDLRKQAFNWGAK.....NGVPTSPSA....
Candida_albicans           274  WGFFQILVSFIVSLIAPIANWFI.....NRDIKNSFKKTKKE
Saccharomyces_cerevisiae   285  WIFGVLSNILVVPFYMVGCSWYIRKWFRENDGKKAN......
Eremothecium_gossypii      268  WLLGALVNLLVVPIYMLLCRWDIHKWRTQREETHLAAKTD..
Homo_sapiens               295  VTMGL..FRTIALFYLGSFDSIVRRCMMQREKSENADKTA..
```

Figure S12: Multiple sequence alignment of *Pf*SgaC (locus ID: KW062_RS19775) with eukaryotic and prokaryotic KDSR homologues.

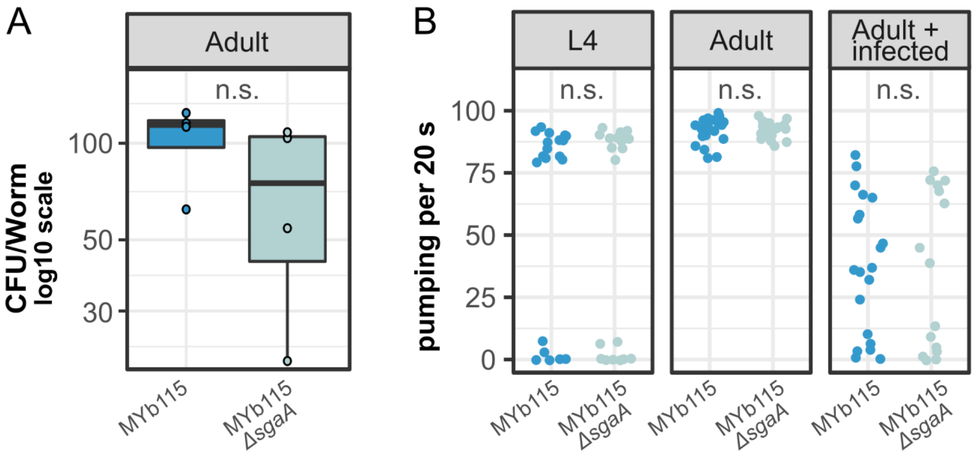

Figure S13: MYb115-derived SLs do not affect host colonisation or *C. elegans* feeding behaviour (A) Bacterial load of adult worms exposed to either MYb115 or MYb115 Δ*sgaA*. No significant difference between the colonisation of the worm between the two bacterial treatments. *t*-test was performed (p = 0.1625). **(B)** Pumping of different worm stages either on MYb115 or MYb115 Δ*sgaA*. No significant difference between the pumping of worms fed with MYb115 or MYb115 Δ*sgaA* depending on the given larval stage/ treatment, pairwise Wilcoxon test was performed *(*p = 1.000).

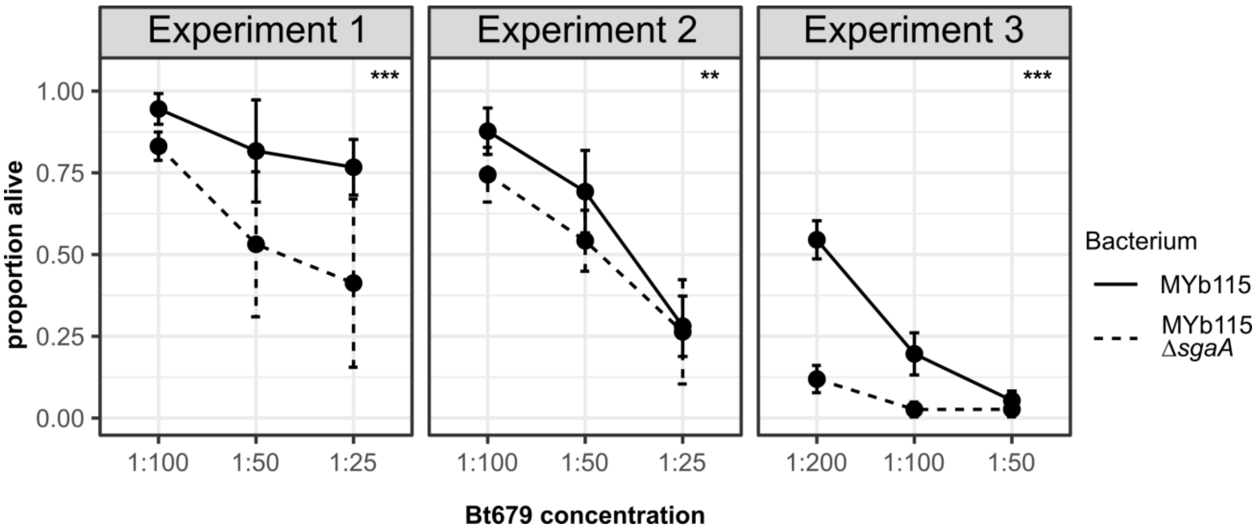

Figure S14: Survival of N2 on MYb115 and MYb115 Δ*sgaA* after infection with *Bacillus thuringiensis* Bt679. Means ± standard deviation (SD) of n = 3, are shown in all survival assays. Statistical analyses were carried out with the GLM framework and Bonferroni adjustment for multiple testing, ***p < 0.001, **p < 0.01, *p < 0.05. All p-values can be found in Supplementary Data 6.

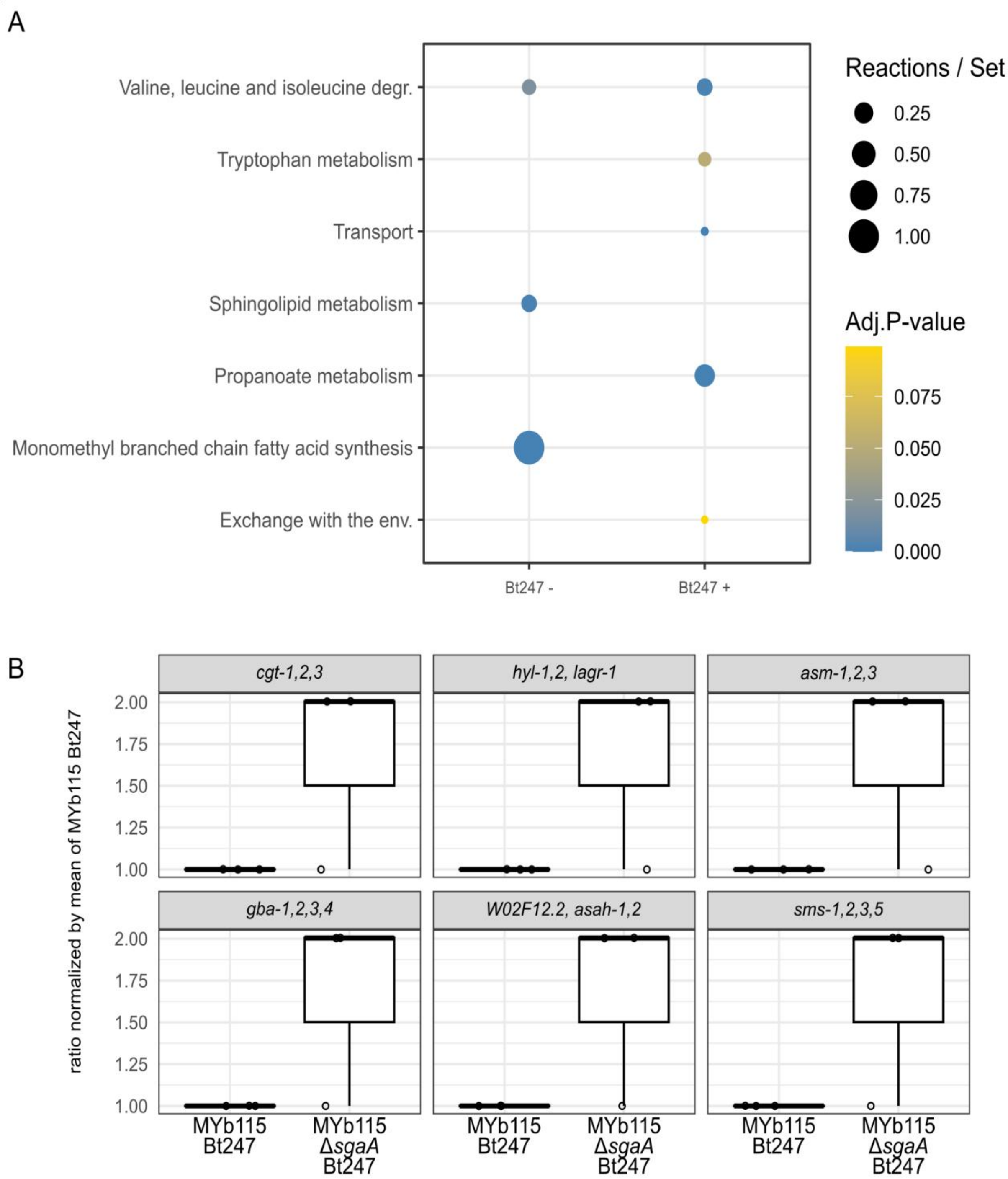

Figure S15: Metabolic network analysis reveals that MYb115-derived SLs affect host fatty acid and SL metabolism. (A) Flux enrichment analysis results in the absence (Bt247-) and presence (Bt247+) of the pathogen. Significant reactions comparing mutant and WT conditions from linear regression models of centre and OFD data layers were combined (while removing duplicates) and used against the background of all reactions within the iCEL1314 model. Enrichment was performed with the FEA function in the COBRA toolbox. **(B)** Ratio of upper bound (ub) values for six reactions encoded by the *C. elegans* SL metabolism enzymes *cgt-1*, *2*, *3*, *hyl-1*, *2* and *lagr-1*, *asm-1*, *2*, *3*, *gba-1*, *2*, *3*, *4*, *W02F12.2* and *asah-1*, *2*, and *sms-1*, *2*, *3*, *5* that all have ceramide as a substrate or product. FVA upper bound values normalised by mean upper bound value of the MYb115_Bt247 group for each reaction.

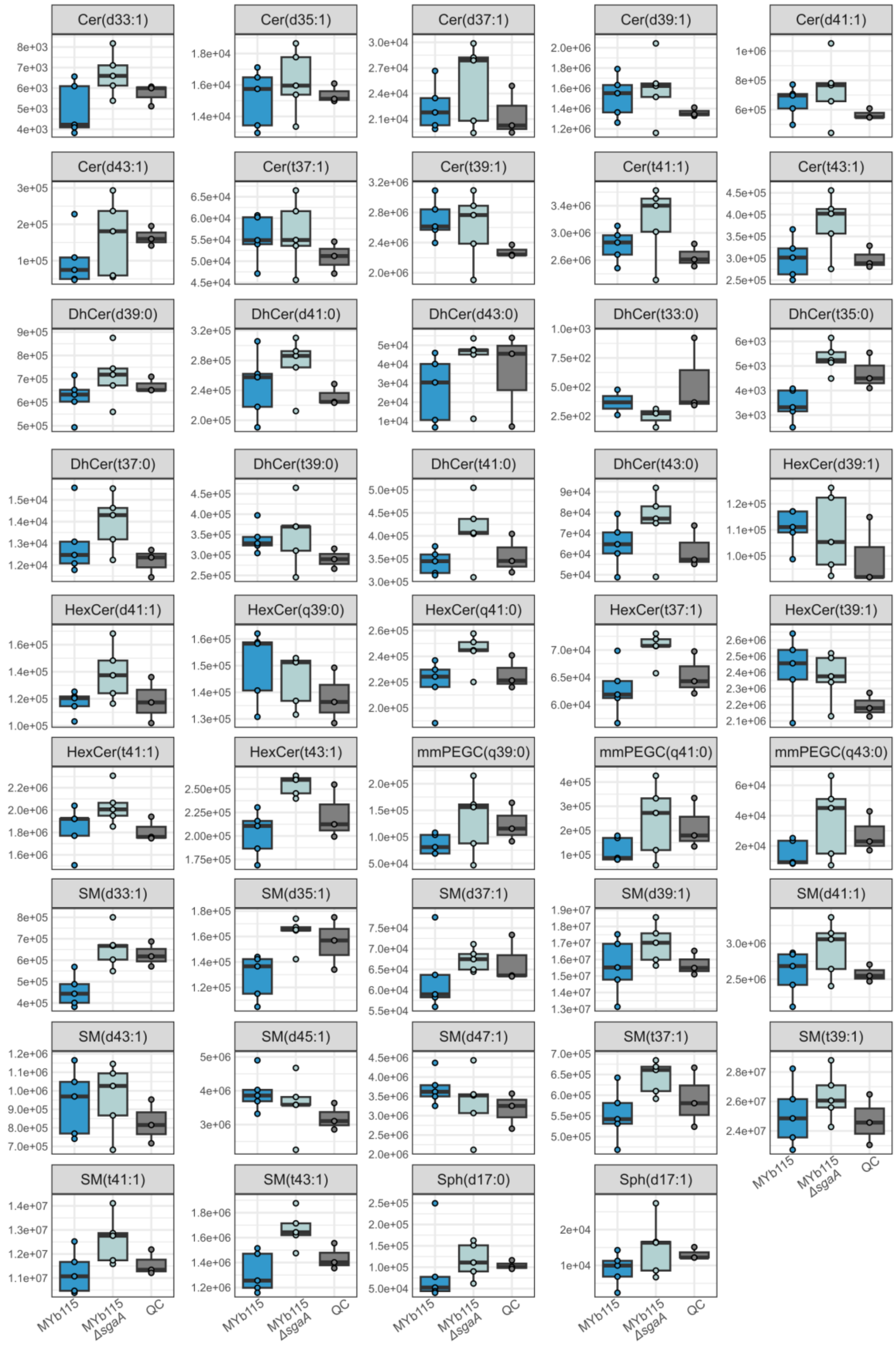

Figure S16: Effects of MYb115-derived SLs on *C. elegans* SL profiles. The boxplots show the difference in ratio of different SLs in worms exposed to MYb115 Δ*sgaA* and MYb115, the data is summarised in the heatmap (**Figure 4B**). Dihydroceramides (DhCer), Ceramides (Cer), Sphingomyelins (SM), Hexosylceramides (HexCer), with hydroxylated fatty acyls (t) or non-hydroxylated fatty acyls (d), Hexosylceramides with phytosphingosine base and hydroxylated fatty acyls (HexCer(q)), monomethyl phosphoethanolamine glucosylceramide (mmPEGC(q)).

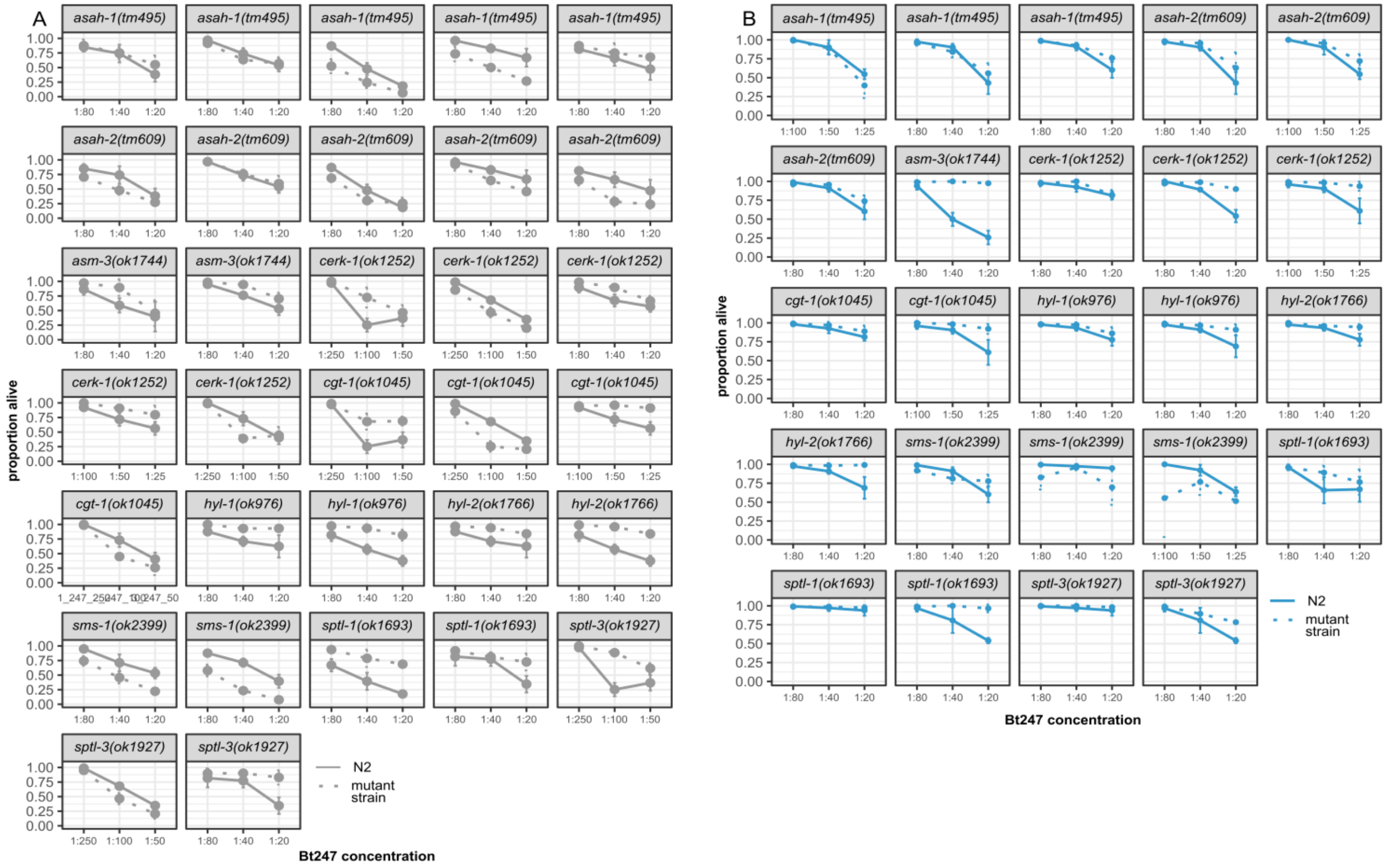

Figure S17: Overview of all individual survival assays from heatmap (Figure 5C), comparing the survival of N2 *versus* the different *C. elegans* SL metabolism mutants on either OP50 **(A)** or MYb115 **(B)**. Means ± standard deviation (SD) of n = 4, are shown in all survival assays. Statistical analyses were carried out with the GLM framework and Bonferroni adjustment for multiple testing, ***$p < 0.001$, **$p < 0.01$, *$p < 0.05$. All p-values can be found in Supplementary Data 12.

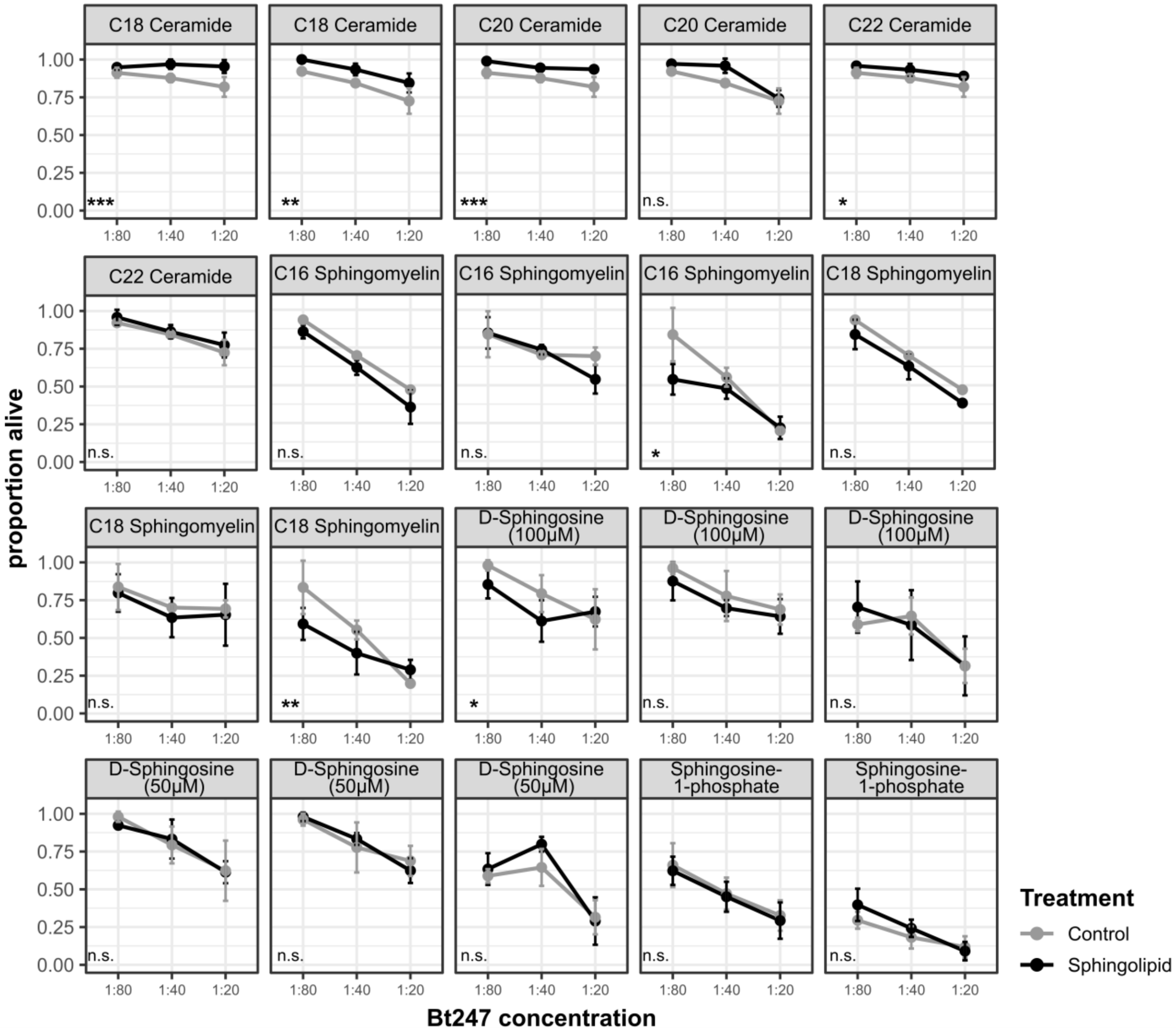

Figure S18: Survival assays of *C. elegans* supplemented with various commercially available SLs. SLs were dissolved in EtOH or MeOH and equal amounts of EtOH or MeOH were used as control treatment in all survival assays. Survival data represent means ± standard deviation (SD) from three independent experiments (n = 3). Statistical analyses were performed using the GLM framework with Bonferroni correction for multiple comparisons (***p < 0.001, **p < 0.01, *p < 0.05). Full *p*-values are provided in Supplementary Data 12**.**

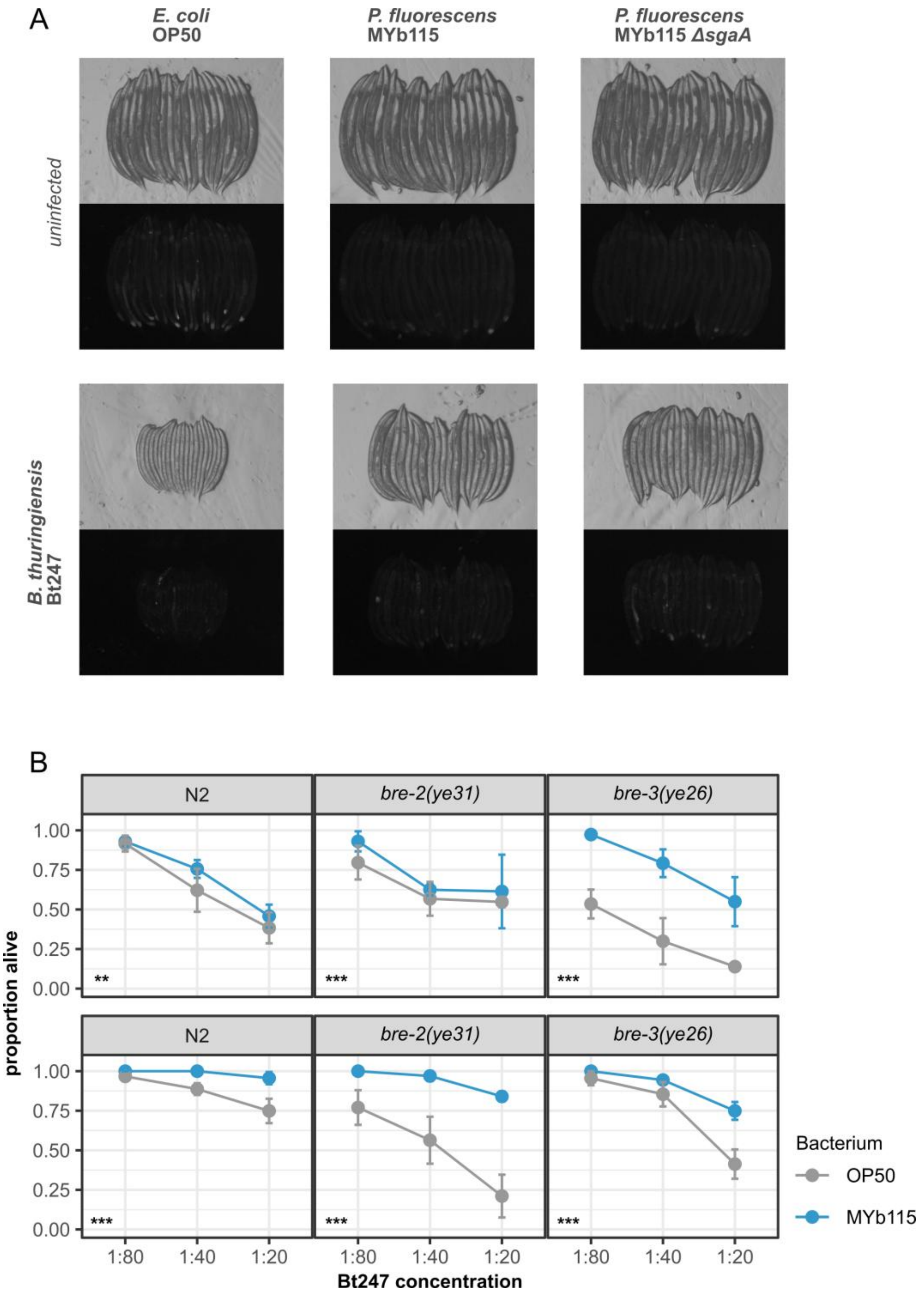

Figure S19: MYb115-mediated protection is independent of the *C. elegans* mitochondrial surveillance response and of complex glycosphingolipids. (A) Light and fluorescence microscopy images of *hsp-6p::gfp* animals raised on either *E. coli* OP50, *P. fluorescens* MYb115 or *P. fluorescens* MYb115 Δ*sgaA* uninfected or infected with Bt247. Images were taken 24h post-infection (worms were 1 day old adults). (B) Survival of *bre*-2(ye31) and bre-3(ye26) glycosphingolipid biosynthesis mutants. Means ± standard deviation (SD) of n = 3 are shown. Statistical analyses were carried out with the GLM framework and Bonferroni adjustment for multiple testing, ****p* < 0.001, ***p* < 0.01, **p* < 0.05. All *p*-values can be found in Supplementary Data 14.

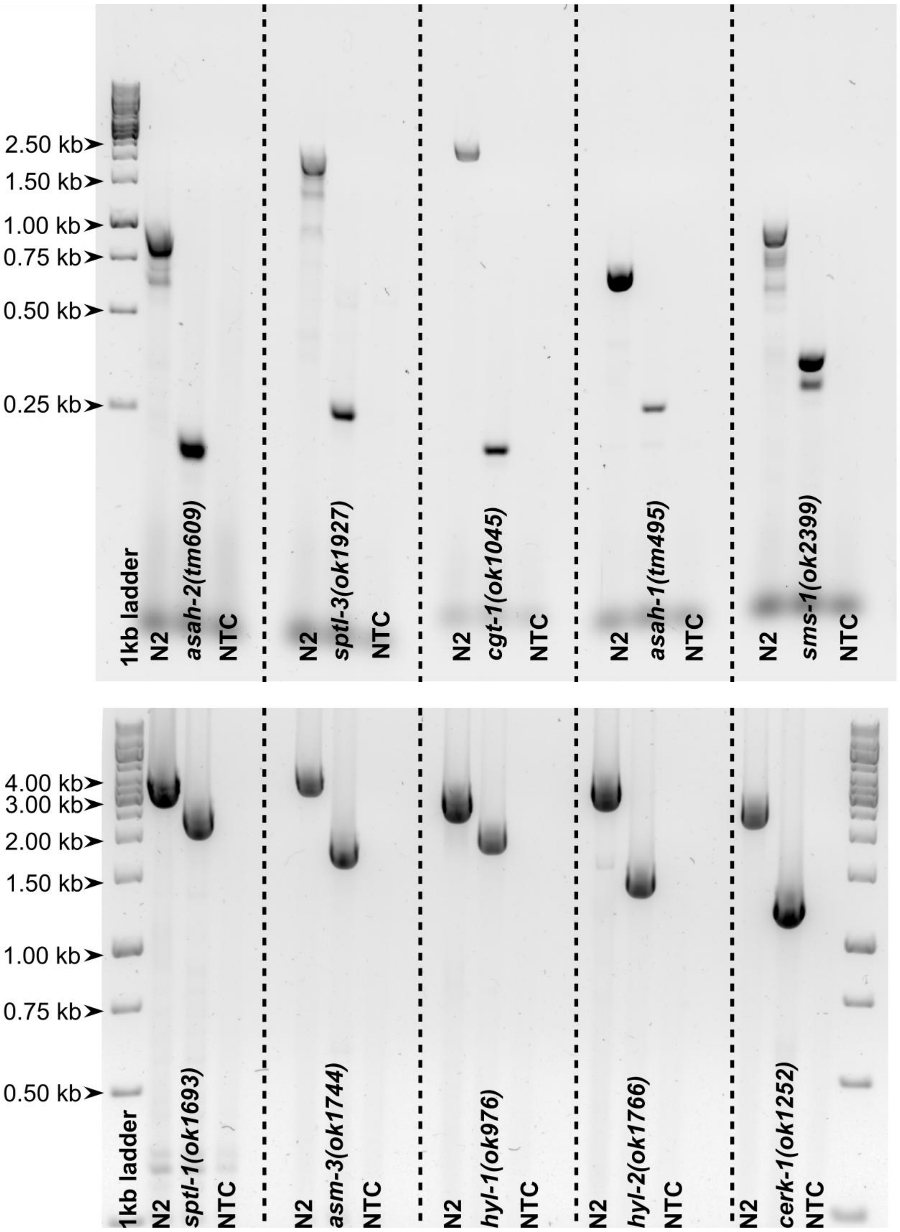

Figure S20: Genotyping of the *C. elegans* SL biosynthesis mutants. Deletion size: *asah-2(tm609)* = 700 bp; *sptl-3(ok1927)* = 1585 bp; *cgt-1(ok1045)* = 1853 bp; *asah-1(tm495)* = 400 bp; *sms-1(ok2399)* = 622 bp; *sphk-1(ok1097)* = 1928 bp; *sptl-1(ok1693)* = 963 bp; *asm-3(ok1744)* = 1558 bp; *hyl-1(ok976)* = 863 bp; *hyl-2(ok1766)* = 1626bp; *cerk-1(ok1252*) = 1300 bp (Supplementary Data 15)

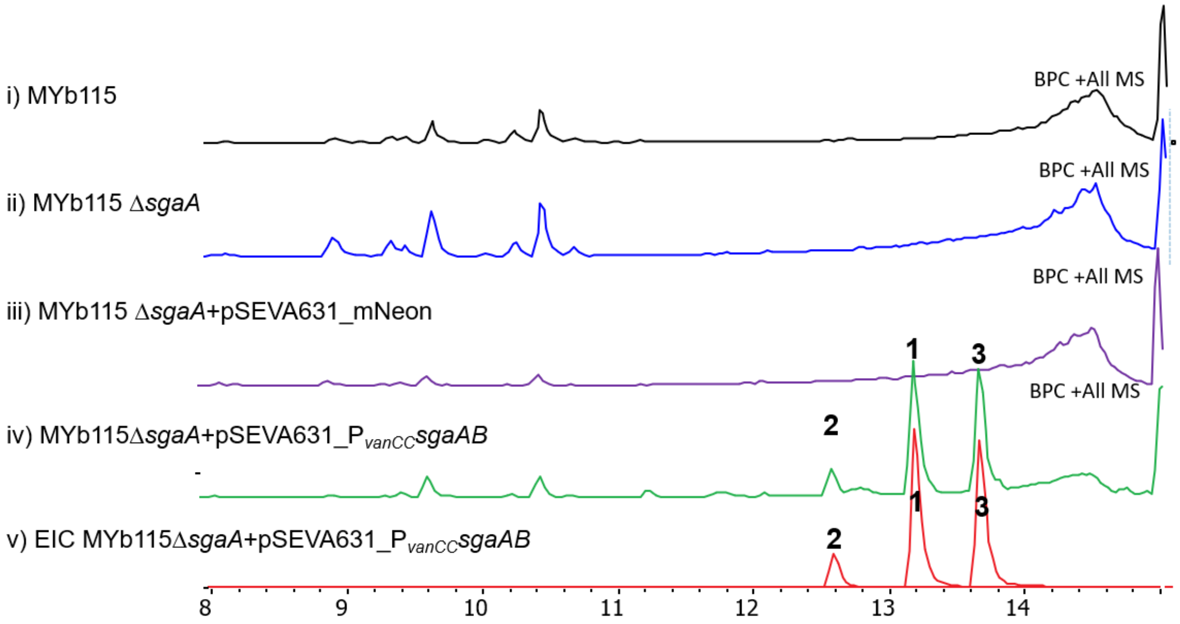

Figure S21: Complementation series of MYb115 Δ*sgaA* and MYb115 Δ*sgaB* using the vanillc aicd inducible P_{vanCC} promoter on the pSEVA631 plasmid. Successful complementation, restoring SL production, required the complete *Pf*SgaAB biosynthetic gene cluster (BGC) on the pSEVA631 plasmid. Only when the complete *PfsgaAB* was co-complemented in MYb115 Δ*sgaA* mutant, the production of all three expected compounds **1** (*m/z* 414.4 $[M+H]^+$), **2** (*m/z* 386.4$[M+H]^+$) and **3** (*m/z* 442.4 $[M+H]^+$) can be detected.
