# Supplementary material for: Polyketide synthase-derived sphingolipids mediate microbiota protection against a bacterial pathogen in C. elegans
Source: Nat Commun. 2025 Jun 3;16:5151. doi: 10.1038/s41467-025-60234-1 (PMC12134224; doi:10.1038/s41467-025-60234-1)
Supplement: Supplementary file 2 — Description of Additional Supplementary Files [file 41467_2025_60234_MOESM2_ESM.docx]

**Description of Additional Supplementary Files**

File Name: Supplementary Data 1

Description: Raw data and results of statistical analyses for survival assays shown in Figure 1 A, B, E, and F and for correlation analysis in Figure 1H

File Name: Supplementary Data 2

Description: Isotopic labeling experiment data, belonging to Figure S1.

File Name: Supplementary Data 3

Description: Relative abundance of the sphinganine compounds 1, 2 and 3 and the PG- sphingolipids 4, 5 and 6 in MYb115 wt and MYb115 PBADsga in the presence of arabinose (ara) for activation or in the presence of glucose (glc) for repression of transcription of PBADsga, belonging to Figure S2.

Sum formula and LC-MS/MS data of MYb115-derived sphinganines 1-3 and phosphoglycerol sphingolipids 4-6 discovered by lipidomic analysis of MYb115. Retention time in minutes (RT [min]); mass-to-charge ration (m/z); theoretical mass (theo), experimental mass (exp); mass error in parts per million (Δppm).

File Name: Supplementary Data 4

Description: Distribution of the two-gene MYb115 PKS SgaAB in bacteria, belonging to Figure 3B

File Name: Supplementary Data 5

Description: Raw data of colonization and feeding assays shown in Figure S13A and S13B, respectively.

File Name: Supplementary Data 6

Description: Raw data and results of statistical analyses for survival assays shown in Figure S14

File Name: Supplementary Data 7

Description: Enrichment analysis data and normalized counts of RNAseq data, belonging to Figure 4

File Name: Supplementary Data 8

Description: Raw data and results of statistical analyses for survival assays shown in Figure 4

File Name: Supplementary Data 9

Description: Raw data of vesicle counts, belonging to Figure 5

File Name: Supplementary Data 10

Description: Metabolic network and pathway enrichment analysis data, belonging to Figure 6A and Figure S15

File Name: Supplementary Data 11

Description: *C. elegans* lipidomics data, belonging to Figure 6B and Figure S16

File Name: Supplementary Data 12

Description: Raw data and results of statistical analyses for survival assays summarized in Figure 7C and shown in Figure S17

Table 13

Description: Raw data and results of statistical analyses for survival assays shown in Figure S18

File Name: Supplementary Data 14

Description: Raw data and results of statistical analyses for survival assays shown in Figure S19B

File Name: Supplementary Data 15

Description: Primer genotyping of C. elegans sphingolipid metabolism mutants, belonging to S20

File Name: Supplementary Data 16

Description: Sequences of sgaAB region in MYb115 wildtype, corresponding region in MYb115 delta sgaA and MYb115 delta sgaB
